# Portable and Digital MOX Sensor Electronic Nose with Thermal Modulation: Design, Stability Analysis, and Long-Term Validation

**DOI:** 10.3390/s26113370

**Published:** 2026-05-26

**Authors:** Víctor González, Juan Álvaro Fernández, Patricia Arroyo, Jesús Lozano

**Affiliations:** Industrial Engineering School, University of Extremadura, 06006 Badajoz, Spain; victorgb@unex.es (V.G.); jalvarof@unex.es (J.Á.F.); parroyoz@unex.es (P.A.)

**Keywords:** temperature-modulated electronic nose, digital MOX gas sensors, chemometrics, machine learning

## Abstract

**Highlights:**

**What are the main findings?**

- A compact e-nose was designed using **modern digital MOX gas sensors** with programmable temperature modulation, which is an improvement over typical analog temperature modulation solutions.
- The system implements **temperature sweeps** that generate multidimensional sensor signatures, enhancing selectivity and chemical information compared to conventional operation.

**What are the implications of the main findings?**

- The device demonstrated **high stability and repeatability**, quantified through RMSE analysis.
- The system successfully discriminated EVOO from pomace oil across multiple days of testing, demonstrating a **robust and portable platform** suitable for quality control and industrial applications.

**Abstract:**

A portable electronic nose based on modern digital metal oxide (MOX) gas sensors and programmable temperature modulation was developed and validated. The system integrates four modern commercially available MOX sensors capable of generating temperature-dependent odor fingerprints and multidimensional sensor responses compared with conventional fixed-temperature operation. The performance of the device was assessed in terms of sensor stability, repeatability, and pattern-recognition capability under long-term operation. As a proof of concept, the electronic nose was applied to the discrimination of Extra Virgin Olive Oil and pomace oil. Repeatability analysis using the Root Mean Squared Error (RMSE) demonstrated stable responses across one month of measurements. Temperature-modulated signals were processed using Principal Component Analysis (PCA) and classified with k-Nearest Neighbors (KNNs) and Multilayer Perceptrons (MLPs), achieving 100% accuracy after selecting the most repeatable sensor. These results highlight the robustness and analytical potential of temperature-modulated digital MOX sensors and demonstrate the feasibility of a compact and highly reproducible electronic-nose platform suitable for complex odor-analysis tasks in real-world applications.

## 1. Introduction

Electronic noses have emerged as powerful analytical tools capable of mimicking the human sense of smell by combining gas sensor arrays with computational models for pattern recognition [1,2]. The most used gas sensors in electronic nose applications are the metal oxide semiconductor sensors (MOX), as they respond very quickly and also have a low price and consumption. These sensors are composed of a thin film of metal oxide materials deposited on a substrate and maintained at a specific operating temperature using a heating element [3]. The measurable parameter in these devices is their electrical resistance, which varies depending on reducing gases or oxidizing gases. The main drawbacks of these types of sensors are the low selectivity and sensitivity in analyzing complex mixtures of volatile compounds, as they react to different gases. To address this issue, **temperature modulation** has become a cornerstone strategy for enhancing the sensitivity and selectivity of MOX-based e-noses [4,5]. The principle behind temperature modulation is that changes in temperature of the heating elements can alter the chemical interactions between the analytes and the sensing materials, producing rich and multidimensional temporal signatures that are not accessible with a single fixed temperature measurement [5].

Several works in recent years have employed temperature-modulated electronic noses, such as the research conducted by Liu et al. [6], where a set of different MOX sensors were used in a modular board for the identification of liquors. Another modular electronic nose was shown in the work by Rescalli et al. [7], where eight analog sensors were used to detect volatile compounds with different temperature patterns; both works made the patterns in long time periods. Other works include the design of an ultra-fast temperature-modulated electronic nose with analog and commercial MOX sensors [8], a designed carbon nanotube-TiO_2_ electronic nose for monitoring VOCs [9], and the use of multifrequency and voltage signals applied to analog sensors heaters [10].

The combination of temperature-modulated sensing and machine learning has therefore positioned modern electronic noses as highly promising platforms for real-world applications requiring reliability, portability, and high discrimination power [11]. Additional studies have developed electronic noses using temperature modulation in Olive Oil applications as olive oil characterization remains a representative case study due to its complex aromatic profile and economic relevance. The works include the use of MQ-series SnO_2_ sensors with an Arduino board, using sinusoidal temperature modulation to classify different kinds of olive oils [12], and also the use of a single analog sensor to very quickly (4 s) measure different mixtures of olive oil [13]. Although many recent works have worked with temperature-modulated MOX sensors, most existing platforms rely on analog and obsolete sensors. And few works have worked with temperature modulation/temperature variations on modern commercially available digital MOX sensors [14,15,16,17,18]. Thus, their integration into electronic nose devices is currently a little-explored field.

In this context, the present work introduces a novel portable electronic nose, integrating temperature-modulated and modern digital MOX gas sensors. The main objective of this work is to present and finally validate a novel portable electronic nose, integrating temperature-modulated and modern digital MOX gas sensors. Unlike conventional electronic noses, the proposed system implements programmable thermal modulation on modern MOX sensors—designed to enhance selectivity and generate richer odor fingerprints that cannot be extracted with conventional electronic nose measurements. This study aims to evaluate the repeatability and stability of the sensors over one month and assess their ability to discriminate Extra Virgin Olive Oil and pomace oils as a demonstrative and challenging test case using machine learning models. Overall, the objective is to demonstrate the feasibility of a compact and highly reproducible electronic nose platform that represents a significant advancement over existing approaches for temperature-modulated electronic noses.

## 2. Materials

### 2.1. Hardware Description

#### 2.1.1. System Architecture

**Figure 1:**
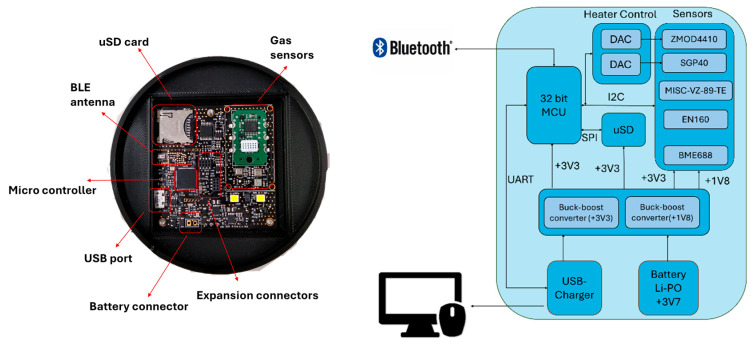
Electronic nose designed (**Left**). Block diagram of the electronic nose (**Right**).

The prototype integrates an olfactory module based on metal oxide (MOX) gas sensors. The main board dimensions are 49 × 51 mm. The electronic nose system developed for this work, along with its block diagram, is shown in Figure 1. The device is composed of different parts, with the main block being the microcontroller. The microcontroller used is an STM32WB55 from STMicroelectronics (Geneva, Switzerland). This chip integrates two cores, a 64 MHz Arm Cortex-M4 and a 32 MHz Arm Cortex-M0+, 1 Mbyte of Flash memory, and a Bluetooth Low Energy (BLE) 5.4 module for wireless communication. The microcontroller is responsible for communicating with other blocks, such as an SD card driver via SPI protocol, and communicating with the different sensors used via I^2^C. Also, it manages data communication, which can be made wirelessly via BLE to a smart device or via USB serial communication to a PC. USB is also used for charging a Li-PO battery, enabling portable operation of the device. In addition, expansion connectors are provided to connect external sensors via I^2^C or SPI and improve future detection capabilities.

#### 2.1.2. Gas Sensor Array

The gas sensor array used includes 5 different MOX gas sensors. Four of the MOX sensors used have the particularity that they can modulate the temperature hotplate, thus allowing temperature sweeping within their operative temperature range. Two of them (BME688 [19] and ENS160 [20]) allow temperature modulation by programming the temperature registers of the hotplates, while the other two (ZMOD4410 [21] and SGP40 [22]) achieve modulation by changing the hotplate voltage using a DAC to regulate it. Signals measured from sensors according to datasheets, and the operative temperature/voltage range, are summarized in Table 1.

**Figure 2:**
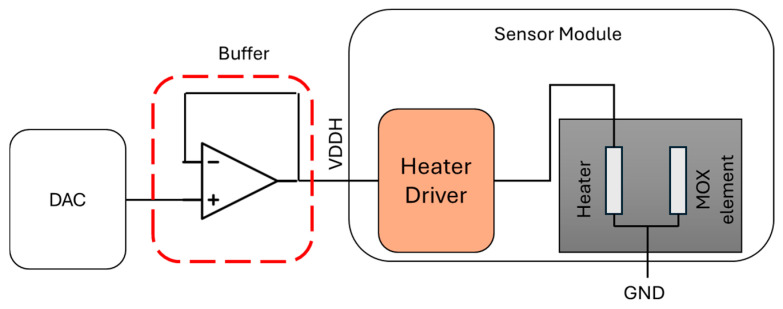
Heater control block diagram.

The way the heater is controlled is shown in Figure 2. Firstly, a programmed voltage is generated with a programmable DAC MCP4725 from Microchip (Chandler, AZ, United States), and the output is buffered with a low output impedance operational amplifier, AD8531 from Analog Devices (Wilmington, MA, United States). Finally, this voltage is applied to the hotplate pad (VDDH), which controls the heater driver applied to the sensor heater.

### 2.2. Firmware Description

**Figure 3:**
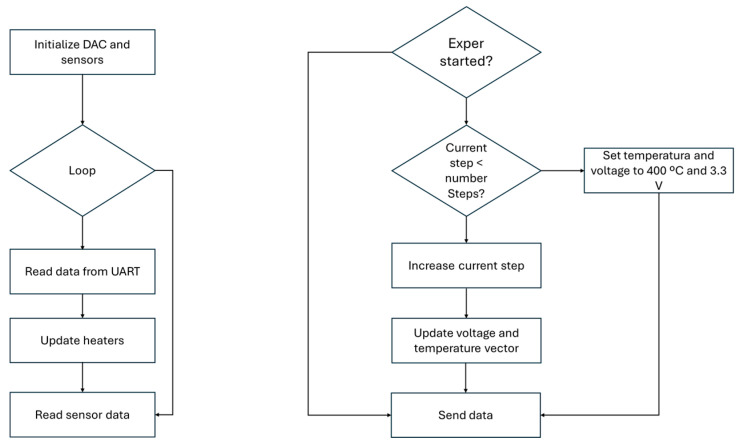
Firmware flow diagram. Main loop (**Left**). Interruption (**Right**).

The prototype is programmed following the flow diagram shown in Figure 3. Firstly, when the device is turned on, there is an initialization stage for all the sensors used, which includes the beginning of communication with the I^2^C bus. Once the initialization is finished, the main loop is responsible for executing the code repeatedly. Here, the device checks if there is any incoming command from the serial port. These commands, summarized in Table 2, include configuration parameters such as the type of temperature sweep and the number of temperature steps, among others. Once the incoming data from the UART is checked, the heaters are updated with the corresponding temperature/voltage values, and finally, the data from the sensors is read.

Parallel to this code, a timer interruption occurs every 3 s; this interruption is responsible for making the temperature/voltage sequence according to the type of sweep selected by the user. In addition, the read data is sent via the serial port in this interruption, ensuring that the data is transmitted every 3 s.

### 2.3. Control Software

**Figure 4:**
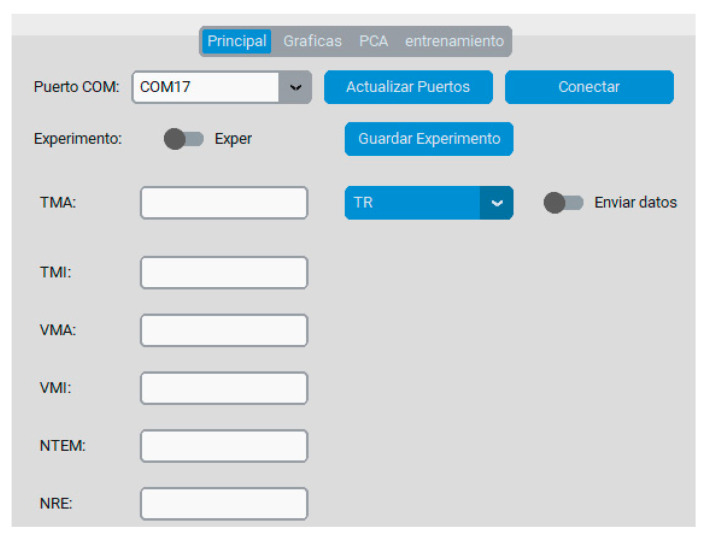
Software main interface.

To operate with the device, a dedicated user interface application was developed in Python 3.13.1 with CustomTkinter 5.5.2 [23]. The main interface is shown in Figure 4. The software is structured into multiple windows: one for experimental configuration, and others for real-time data visualization.

The experimental configuration window (Figure 4) enables device selection via a COM port and provides control over several parameters, including the applied temperature profile (square wave, sawtooth wave, and triangle wave), and specific temperature settings such as maximum/minimum temperature/voltage, the number of steps of temperatures/voltages, and also the number of cycles performed. In addition, data can be saved for future data analysis.

### 2.4. Sample Preparation

Extra Virgin Olive Oil (EVOO) and olive pomace oil samples were used; 10 mL samples from the same bottles were acquired each day on 9 different days distributed over 1 month, as can be seen in Table 3. For each day, EVOO and pomace samples were measured four times consecutively, resulting in 36 samples measured for each kind of olive oil sample analyzed to evaluate the repeatability of the measured responses.

## 3. Methods

### 3.1. Experimental Protocol

**Figure 5:**
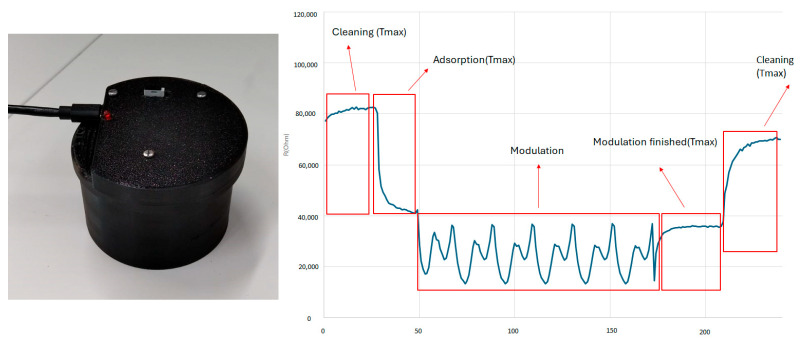
Measurement process with the device.

Gas sensor measurements consisted of static headspace analysis, made on white cylindrical PLA containers with a 75 mm diameter and 45 mm height. The whole measurement process is explained below, and the setup as well as the measurement process are shown in Figure 5:

Adsorption (2.5 min): For each new olive oil sample, an adsorption period is required to ensure a stable baseline and a uniform concentration of volatile compounds in the static headspace. During this phase, the sensor’s heaters were kept at maximum temperature (400 °C) and voltage (3.3 V).

Modulation (8 min): In this phase, the temperature pattern was applied, and the process was repeated to ensure repeatability for four consecutive cycles. The temperature pattern consisted of a triangular waveform that decreased from 400 °C to 200 °C/3.3 V to 0.5 V and increased to 400 °C/3.3 V (coinciding with the operative range to account for full response) with 21 steps for the decreasing/increasing stage; thus, a total of 41 temperature steps with +/−10 °C increment/decrement were performed.

Cleaning (6 min): After the patterns were finished, the heaters were brought back to maximum temperature/voltage, and the electronic nose was exposed to a bottle with ambient air conditions to ensure cleaning of the sensor’s surface.

Additionally, a preliminary comparative evaluation between triangular, square, and sawtooth modulation strategies was conducted under identical experimental conditions. This exploratory analysis was performed to qualitatively assess the influence of waveform shape on signal quality.

### 3.2. Data Analysis

#### 3.2.1. Gas Sensor Feature Extraction

Resistance responses across the temperature sweep were used (eliminating the MISC-VZ-89-TE response as it cannot modulate its temperature hotplate) as they represent the raw data without any preprocessing. Thus, for each resistance measure (19) and each cycle performed, a total number of 41 characteristic values were obtained, coinciding with the number of temperature steps.

#### 3.2.2. Prototype Matrix

For each day and olive oil type, four measurements were taken, resulting in 36 samples for the two kinds of olive oil. Thus, a prototype matrix of 19 × 72 × 41 was obtained, where the first dimension is the number of sensors (gas resistance), the second one (number of rows) is the samples taken, and the third one (columns) is the characteristic variables (response of the sensors to temperature steps) of each cycle performed on each sample. Thus, concatenating the response of all the sensors, the final prototype matrix is 779 × 72.

#### 3.2.3. Repeatability Analysis

To check whether a sensory system can perform repeatable measurements, it is necessary to define appropriate metrics [24] that allow the expression of the similarity between two vectors. For all the cycles measured on all the days, the repeatability of the gas sensors was measured. To quantify repeatability measurements, the RMSE was used (Equations (1)–(3)). The advantage of using the RMSE is that the calculated value is in the same unit as the original magnitude measured.

(1) $$\;\;\;\;\;\;\;\;\;\;\;\;\;\;\;\;\;\;\;\;\;\;{RMSE}_{C_{x}}=\sqrt{\frac{1}{n}\sum_{i=1}^{n=41}{\left(c_{x_{i}}-Z_{i}\right)}^{2}}$$

(2) $$\;\;\;\;\;\;\;\;\;\;\;\;\;\;\;\;\;\;\;\;\;\;\;\;Z_{i}=\frac{1}{m}\sum_{x=1}^{m=36}C_{x_{i}}$$

(3) $$RMSE=\frac{1}{m}\sum_{x=1}^{m=36}{RECM}_{C_{x}}$$

where *n* = 41 is the number of samples/steps taken each cycle $c_{x}$ measured, *m* = 36 is the number of cycles (samples) measured for each olive oil class, and *Z* is the average calculated from the cycles measured each day. Finally, a global RMSE is calculated to account for the average deviation of the cycles measured.

#### 3.2.4. Temperature Analysis

To check which temperature range is more important, dimensionality reduction of each sensor’s measurements was made using PCA. PCA is a dimensionality reduction algorithm. Its goal is to reduce the data to a few characteristic dimensions that retain most of the information (variance) contained in the original variables. This is achieved by computing new variables (principal components, PCs) from linear combinations of the original variables. These PCs are chosen so that the important information in the data is mainly retained in some of these new variables, thus condensing the information of the samples or observations [25].

Before PCA was made, a standardization of the data was made according to Equation (4):

(4) $$z=\frac{x-u}{s}$$

where *x* is the raw data, and u and s are the mean and the standard deviation. For this analysis, dimensionality reduction was used for the first two PCs, and for each variable (response of each sensor for each temperature), it was studied in the 200–300 °C, 250–350 °C, 300–400 °C and 200–400 °C temperature sweep ranges to account for the effects of how sensors respond to different temperature regions.

#### 3.2.5. AI Analysis

For the classification of the two olive oil classes, k-Nearest Neighbor (KNN) models and Multilayer Perceptrons (MLPs) were selected, using the Scickit Learn library v1.7.2 [26]. The AI technique known as k-Nearest Neighbors (kNNs) classifies an input value by calculating the distances between that value and all the training samples. Among these distances, the *k* training samples closest to the input value are selected, and the input is assigned to the majority class among those *k*-nearest samples [27]. MLP is a type of Artificial Neural Network composed of at least three layers of neurons, where each neuron is not connected to neurons in its own layer but rather to all neurons in the previous layer (input) and to all neurons in the next layer (output). The network is divided into an input layer, which contains the input variables; one or more hidden layers, responsible for processing or separating the information; and an output layer, which contains the output variables corresponding to the different classes to be classified. In each neuron of the hidden layer(s) and the output layer, a numerical value is computed as a linear combination of the inputs weighted by a set of coefficients, to which an activation function is subsequently applied [28].

**Figure 6:**
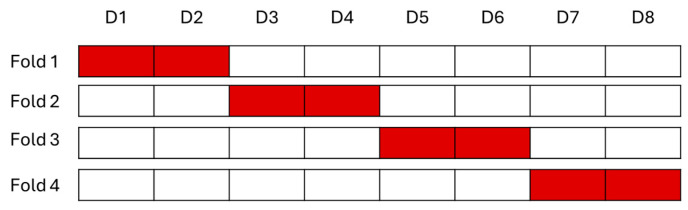
Dataset folds. Test data (red), training data (white).

Data was validated with KNN with parameter K = 3 and MLP with one single hidden layer of 40 neurons and an identity activation function, creating a data set as shown in Figure 6. As can be seen, four different data folds have been created in the first eight days; the first fold takes data from the first two days as a test set and the remaining data as a training set. The subsequent folds displace training data until the last days are included. A final validation of the models calculated on each fold was made, reserving the D9 data as it was one month later than the other data. With this validation method, it is intended to account for the repeatability of the models across time.

To evaluate the quality or suitability of the model, precision was used as the main metric. Precision is defined as the ratio of correct positive predictions (true positives) to the total number of positive predictions made by the model (true positives + false positives).

Dimensionality reduction of the prototype matrix was made using PCA, and before PCA, data was standardized using Equation (4). Firstly, the training data was fitted, and after that, the test data was standardized. Similarly, PCA was made, training data was fitted, and finally, the testing data was transformed to the new space of principal components calculated, avoiding data leakage. Firstly, all the data from the sensors was reduced to the first ten PCs, and finally, less repeatable sensors were eliminated, calculating a new reduced matrix with just three PCs; the number of PCs selected in both cases was chosen to obtain above 95% of the variance of the data.

## 4. Results and Discussion

### 4.1. Comparative Waveform Evaluation

**Figure 7:**
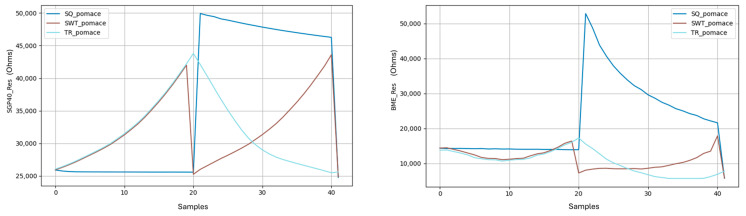

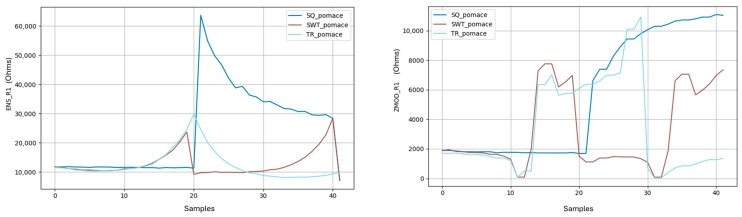
Comparison between the influence of triangular (TR), sawtooth (SWT) and square (SQ) waves on sensors’ response to pomace oil.

A preliminary comparison between triangular, square, and sawtooth temperature modulation strategies was conducted to evaluate the influence of waveform shape on sensors. Figure 7 shows representative response cycles obtained from sensors’ response during the measurement of pomace olive oil using the three modulation profiles.

As observed in the experimental responses, the triangular modulation produces smoother and more continuous transitions during both the heating and cooling stages of the temperature sweep. In contrast, square and sawtooth modulation profiles generate more abrupt variations in the sensor response due to the sudden temperature transitions between minimum and maximum operating values. These effects are particularly noticeable at the transition regions of the modulation cycle, where larger instantaneous response variations are observed for square and sawtooth waveforms. In addition, from a signal-processing perspective, the triangular modulation enables continuous bidirectional sweeps (heating and cooling), generating more extended information than sawtooth and square, increasing the dimensionality of the sensor signature compared to discontinuous modulation strategies.

### 4.2. Repeatability Analysis

**Figure 8:**
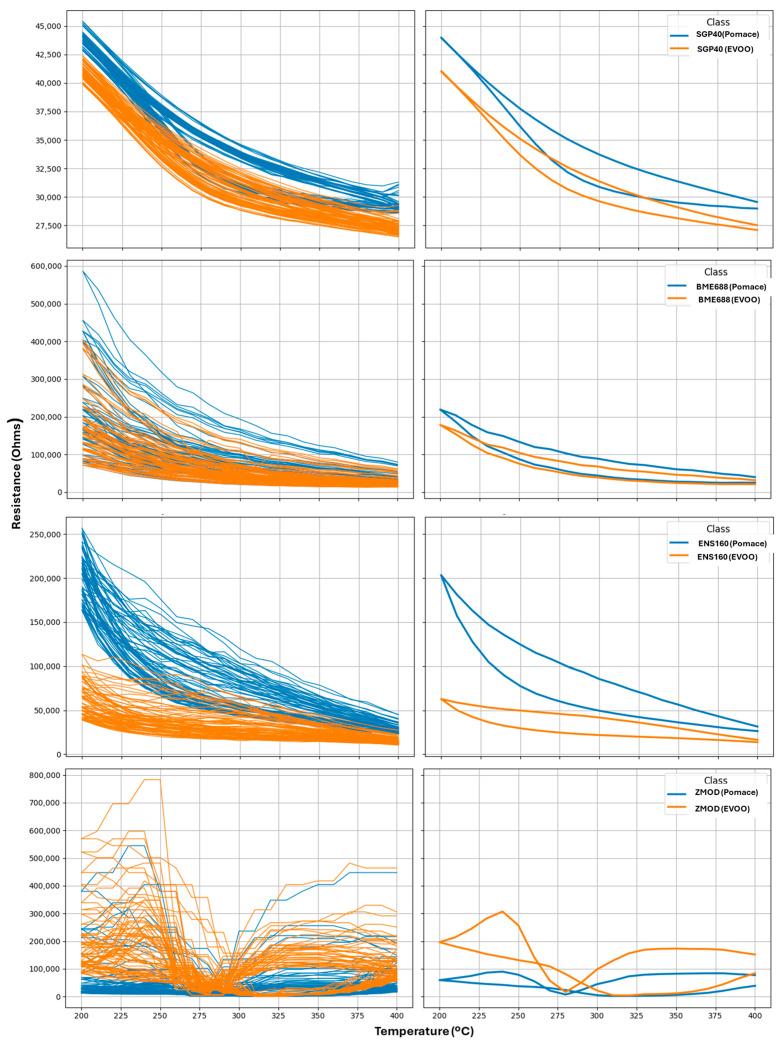
Response of the samples of EVOO and pomace on all the days measured (**left**) and the average of the classes (**right**) corresponding to sensor response of SGP40, BME688, ENS160 (R1), and ZMOD4410 (R1) from top to bottom.

The daily response of the gas sensors to pomace and EVOO, and the average value across triangle temperature sweep as explained in Section 3.1, is shown in Figure 8. The left panel shows each cycle performed for each olive oil type analyzed across the test month (36 cycles for each olive oil class). On the other hand, the panel on the right shows the average value of all cycles measured for each olive oil class.

As can be seen, each sensor responds differently to each olive oil sample analyzed, as temperature modulation analysis accounts for dynamic response of the sensors depending on temperature, creating different odor fingerprints that depend on the temperature sweep. It is also observed that sensors do not react symmetrically to the ascending/descending sweeps; thus, the chemical interaction between olive oil samples and hotplates differs during the temperature sweep.

The RMSE calculated from all the sensors to account repeatability (calculated with Equations (1)–(3)) shows that the most reliable sensors are SGP40 and ENS160, while the least repeatable are ZMOD4410 and BME688. This is demonstrated by the standardized (calculated with Equation (4) to scale the data) and average RMSE calculated for each sample in Table 4.

### 4.3. Temperature Analysis

**Figure 9:**
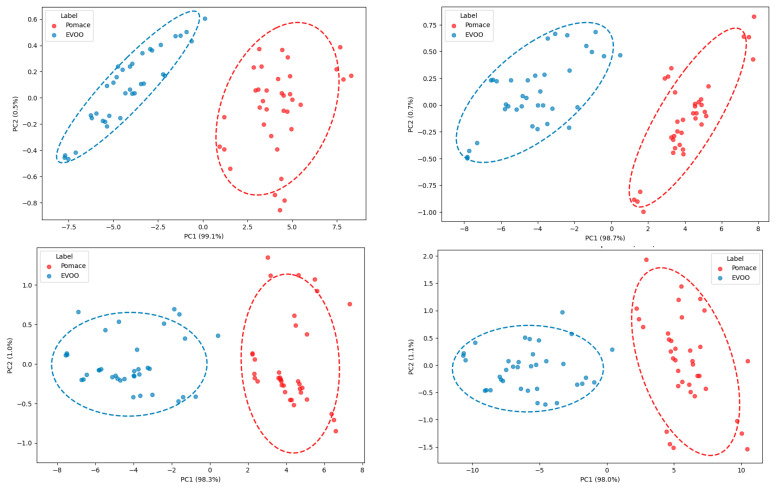
PCA plot of SGP40 response at 200–300 °C range (**Left-Up**). PCA plot of SGP40 response at 250–350 °C range (**Right-Up**). PCA plot of SGP40 response at 300–400 °C range (**Down-Left**). PCA plot of SGP40 response at 200–400 °C range (**Down-Right**).

**Figure 10:**
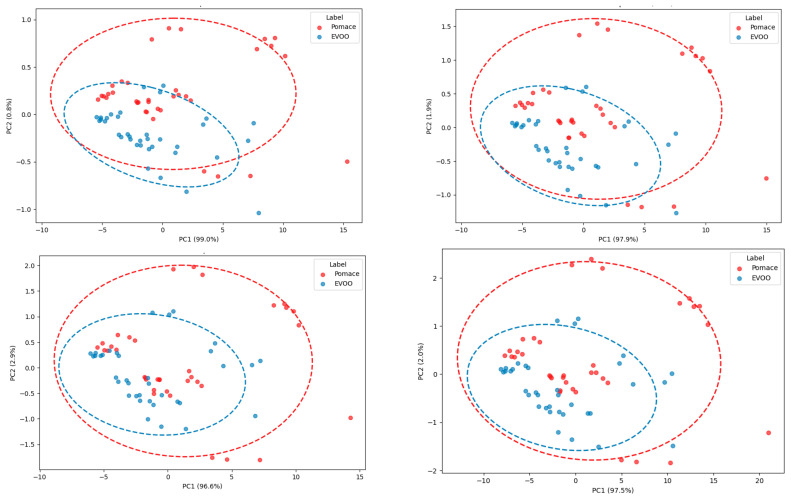
PCA plot of BME688 response at 200–300 °C range (**Left-Up**). PCA plot of BME688 response at 250–350 °C range (**Right-Up**). PCA plot of BME688 response at 300–400 °C range (**Down-Left**). PCA plot of BME688 response at 200–400 °C range (**Down-Right**).

**Figure 11:**
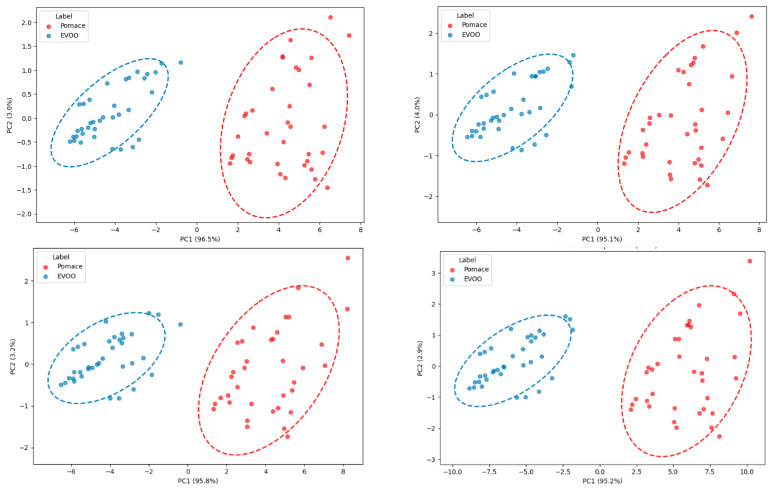
PCA plot of ENS160 (R1) response at 200–300 °C range (**Left-Up**). PCA plot of ENS160 (R1) response at 250–350 °C range (**Right-Up**). PCA plot of ENS160 (R1) response at 300–400 °C range (**Down-Left**). PCA plot of ENS160 (R1) response at 200–400 °C range (**Down-Right**).

**Figure 12:**
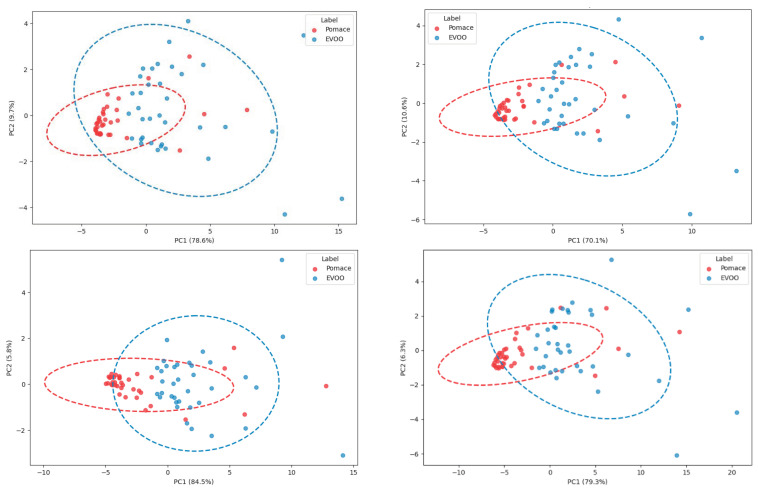
PCA plot of ZMOD4410 (R1) response at 200–300 °C range (**Left-Up**). PCA plot of ZMOD4410 (R1) response at 250–350 °C range (**Right-Up**). PCA plot of ZMOD4410 (R1) response at 300–400 °C range (**Down-Left**). PCA plot of ZMOD4410 response at 200–400 °C range (**Down-Right**).

Figure 9, Figure 10, Figure 11 and Figure 12 present the PCA plots obtained from the sensor responses using triangular temperature sweeps in the ranges explained in Section 3.2.4. As observed in the PCA score plots, a clearer class separation is achieved when using the responses from SGP40 and ENS160 sensors, whereas BME688 and ZMOD4410 exhibit poorer discrimination capability. In addition, no significant differences in class separation are found between the evaluated temperature intervals. This behavior suggests that the discriminatory information is not concentrated within a specific temperature region, but rather distributed throughout the thermal sweep. Therefore, both low- and high-temperature regions contribute to the overall classification performance, supporting the use of the complete temperature range for sensor characterization and validation of the proposed electronic nose platform.

### 4.4. AI Results

**Figure 13:**
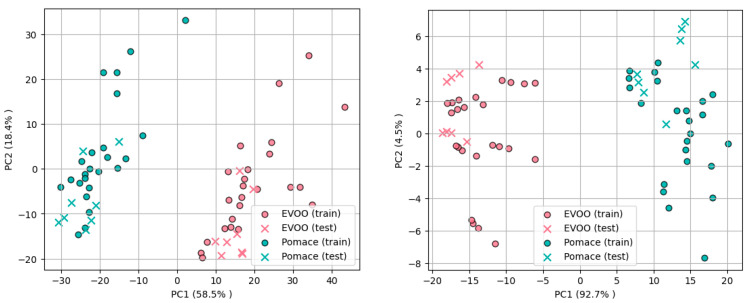
2PCA separating training and test data in fold 1. Considering data from all sensors (**Left**) with 10 PCs. Eliminating ZMOD4410 and BME688 response (**Right**) with 3 PCs.

**Figure 14:**
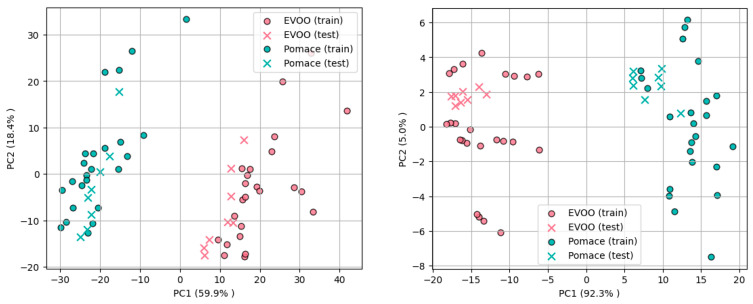
2PCA separating training and test data in fold 2. Considering data from all sensors (**Left**) with 10 PCs. Eliminating ZMOD4410 and BME688 response (**Right**) with 3 PCs.

**Figure 15:**
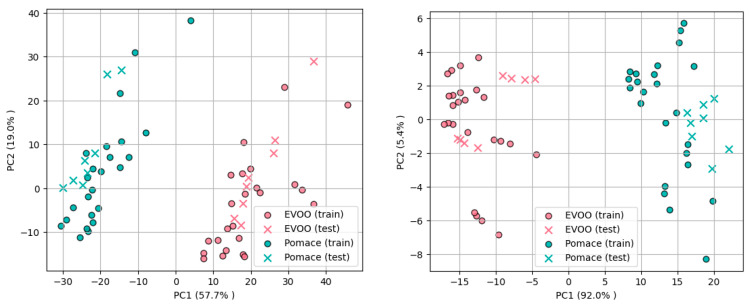
2PCA separating training and test data in fold 3. Considering data from all sensors (**Left**) with 10 PCs. Eliminating ZMOD4410 and BME688 response (**Right**) with 3 PCs.

**Figure 16:**
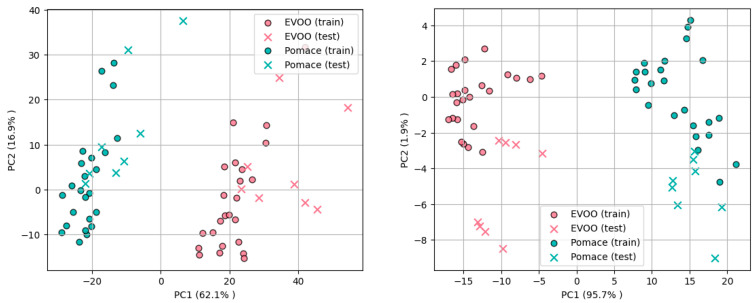
2PCA separating training and test data in fold 4. Considering data from all sensors (**Left**) with 10 PCs. Eliminating ZMOD4410 and BME688 response (**Right**) with 3 PCs.

PCA results for the sensor data were first calculated, considering all sensor responses, and then the responses of the less repeatable sensors were eliminated according to Section 4.2 (ZMOD4410 and BME688); also, temperature sweep data was considered from the 200–400 °C range. Figure 13, Figure 14, Figure 15 and Figure 16 show the 2D PC representations for each data fold analyzed. As can be seen, the 2D projection of the two datasets shows a clear separation between the two kinds of olive oil samples. In addition, it can be shown that the information represented in the first two PCs is higher when the ZMOD4410 and BME688 are eliminated; thus, a minor number of PCs is needed to account for the maximum variance of the data.

Validation results of the system according to the folds explained in Section 3.2 are shown in Table 5. It is observed that the overall precision of KNN and MLP models, considering all the data and eliminating data from ZMOD4410 and BME688, is 100% except for fold 2 using all the data with MLP (87.5%).

Once the system has been validated in the first eight measurement days, the models calculated have been tested with data from D9; the results are shown in Table 6. The results show a clear difference between the most repeatable data and the least repeatable. Considering all the sensors, results show a poor precision with both MLP and KNN (50%). However, 87.5% precision was obtained with MLP, evaluating fold 3, as it takes training data from intermediate days between validation and D9 testing. Finally, when ZMOD4410 and BME688 were not considered, 100% precision was obtained.

## 5. Conclusions

In this work, a portable electronic nose integrating temperature-modulated and modern digital MOX gas sensors was presented, and the stability and repeatability of sensors were evaluated with extra Virgin Olive Oil (EVOO) and pomace olive oil samples. Finally, discrimination between these two classes was performed as a first approach for olive oil discrimination.

A preliminary comparative evaluation between triangular, square, and sawtooth modulation strategies, as shown in Section 4.1, indicated that triangular modulation provided smoother responses under the evaluated operating conditions, supporting its selection for the long-term validation protocol.

The experimental results collected over a period of one month demonstrate that the system exhibits high stability and repeatability, particularly for the ENS160 and SGP40 sensors, as evidenced by the RMSE analysis in Section 4.2. In addition, temperature-modulated data successfully highlighted consistent olfactory differences between EVOO and pomace oil, confirming that subtle variations in the olfactory profiles can be exploited for classification purposes.

Furthermore, sensors were also tested in different temperature ranges, as was shown in Section 4.3, concluding that there is not a special range that enhances classification, thus the whole temperature range of the sensors was selected for classification purposes.

From a data analysis perspective, the fusion of signals through dimensionality reduction (PCA) and machine learning (KNN and MLP) revealed strong discrimination capabilities. Although the folds tested achieved a 100% classification considering all the sensors except for fold 2 using MLP (87.5%), removing the least repeatable sensors (ZMOD4410 and BME688) not only reduced the dimensionality requirements but also improved the average classification of the different folds analyzed a month later, achieving 100% correct classification with only three principal components. These results underscore the benefit of data-driven sensor selection and the robustness of the combined sensing approach.

Overall, the performance and portability of the device represent an advancement on temperature-modulated electronic noses designs, as the state-of-the-art shows that analog and obsolete sensors are usually used. On the other hand, classification results demonstrate that integrating a temperature-modulated electronic nose yields a compact and highly reliable solution for olive oil identification, and it supports the potential for real-world applications in olive oil mills, packaging industries, and quality control laboratories, where repeatable and stable analyses are essential.

## 6. Future Works

Based on the results of this study, several promising research directions can further enhance the performance, robustness, and applicability of the electronic nose system:

1. Expansion of the Dataset: Future studies should incorporate a wider variety of olive oil samples, including different cultivars, geographical origins, harvest seasons, and production methods.
2. Detection and Quantification of Adulteration: A key industrial challenge lies in identifying fraudulent mixtures of EVOO with refined or seed oils. Controlled adulteration experiments at different mixing ratios (e.g., 1–50%) would allow for testing the limits of gas sensors.
3. Integration of an Electronic Tongue (e-tongue) and Electronic Eye (e-eye): Incorporating electrochemical or potentiometric taste sensors would complement the olfactory and visual modalities, creating a fully multimodal sensing platform. This additional dimension could significantly enhance the discrimination of oils with similar aromatic profiles but different chemical compositions. In addition, the addition of colorimeters or cameras would account for chromatic differences between olive oil samples and for classification. Thus, the addition of different sensing methods would allow for complementing the insufficiencies between them individually.
4. On-device Machine Learning: Implementing optimized AI models directly on the microcontroller would enable real-time classification without dependence on an external computer. This would greatly improve portability and suitability for field operation.
5. Optimization of the Measurement Protocol: The current measurement cycle requires over 15 min per sample. Future work may explore shortened or adaptive temperature waveforms, feature selection, or rapid-sampling strategies that maintain classification precision while substantially reducing acquisition time. Also, different temperature patterns can be applied to obtain different profiles that could not be achieved by a single pattern.

Collectively, these research directions represent important steps toward transforming the current prototype into a mature, industry-ready tool for the authentication and quality control of food products.

## Figures and Tables

**Table 1:** Gas sensor array.

Sensor	Type	Temperature Regulation	Signals
BME688	MOX	Software (200–400 °C)	Temperature (°C), Relative Humidity (%), Pressure (Pa), Resistance (Ohms)
ENS160	MOX	Software (200–400 °C)	Four Resistances (Ohms), TVOCs (ppb), eCO_2_ (ppm), AQI
SGP40	MOX	Hardware (0–3.3 V)	Resistance (Ohms), VOC_Index
ZMOD4410	MOX	Hardware (0–3.3 V)	13 Resistances (Ohms), TVOCs (mg/m^3^), eCO_2_(ppm), IAQ, EtOH (ppb)
MISC-VZ-89-TE	MOX	-	Resistance (Ohm), eCO_2_ (ppm), TVOCs (ppb)

**Table 2:** Commands read by the device.

Command	Description
EXPER	Start the experiment.
TR	Select a triangular waveform.
SQ	Select square waveform.
SWT	Select the sawtooth waveform.
TMA	Maximum temperature of the sweep.
TMI	Minimum temperature of the sweep.
VMA	Maximum voltage of the sweep.
VMI	Minimum voltage of the sweep.
NTEM	Number of temperatures of the sweep performed.

**Table 3:** Measurements schedule.

Identifier	Date	Number of Consecutive Experiments
D1	16 July 2025	4
D2	17 July 2025	4
D3	18 July 2025	4
D4	21 July 2025	4
D5	22 July 2025	4
D6	23 July 2025	4
D7	24 July 2025	4
D8	25 July 2025	4
D9	18 August 2025	4

**Table 4:** Average RMSE for the different gas sensors.

Variable	RMSE (EVOO)	RMSE (Pomace)
SGP40	0.37	0.29
BME688	0.64	0.91
ENS160_R1	0.31	0.4
ENS160_R2	0.3	0.41
ENS160_R3	0.3	0.42
ENS160_R4	0.19	0.3
ZMOD4410_R1	0.79	0.52
ZMOD4410_R2	0.79	0.54
ZMOD4410_R3	0.81	0.57
ZMOD4410_R4	0.8	0.59
ZMOD4410_R5	0.83	0.62
ZMOD4410_R6	0.86	0.66
ZMOD4410_R7	0.89	0.69
ZMOD4410_R8	0.88	0.72
ZMOD4410_R9	0.88	0.72
ZMOD4410_R10	0.86	0.73
ZMOD4410_R11	0.87	0.73
ZMOD4410_R12	0.84	0.73
ZMOD4410_R13	0.86	0.72

**Table 5:** Precision obtained from the classification models.

Fold	Precision MLP (%). All Data	Precision KNN (%). All Data	Precision MLP (%). No ZMOD4410	Precision KNN (%). No ZMOD4410
1	100	100	100	100
2	87.5	100	100	100
3	100	100	100	100
4	100	100	100	100

**Table 6:** Precision obtained from D9 validation.

Fold	Precision MLP (%). All Data	Precision KNN (%). All Data	Precision MLP (%). No ZMOD4410 and BME688	Precision KNN (%). No ZMOD4410 and BME688
1	50	50	100	100
2	50	50	100	100
3	87.5	50	100	100
4	75	50	100	100

## Data Availability

Data is available at the following URL: https://data.mendeley.com/datasets/mhwf9jnch9, accessed on 15 April 2026.
